# Foreign Medical and Physical Science and Literature

**Published:** 1819-05

**Authors:** 


### FOREIGN MEDICAL AND PHYSICAL SCIENCE AND LITERATURE.

### *Exposition of the Doctrine of* M. Broussais.

(*Continued from p.* 341.)

ALL men have an inclination to form *exclusive* opinions. This disposition, which we have to attribute as general and applicable to all cases, the rules deduced from facts being either too few in number or but imperfectly observed, is one of the results of the inclination of the human mind to disembarrass itself of the painful study of *exceptions*. But, if authors, led on by their ideas, often commit this fault, critics likewise do not always avoid it, and that from the same cause. It is more easy to say that such a writer can see nothing but inflammation, that he treats all diseases by debilitants, and particularly by blood-letting, than to follow him step by step, and study with him the numerous modifications that he adduces of general principles that it may be necessary to establish in individual cases. Such, however, has been the idea which the authors of some new works have wished to give of the doctrine of M. Broussais. This physician has nevertheless endeavoured to determine the cases where he believes tonics may be proper, and he has even essayed to indicate where emetics may be exhibited without danger. In typhus arising under the most debilitating influences, and produced by the action of deleterious miasmata, stimulation is often indispensable; but it ought always to be exerted on the organs the least irritated, so as to avoid the destruction of those which are the seat of congestion. "The circumstances," observes M. Broussais, "where wine and other stimulants may be given inwardly are—1°, when general debility and stupor present themselves with a tongue but slightly red, and without any sign of inflammation in either of the three great cavities; 2°, when these means, instead of rendering the tongue dry and crusted, the thirst more ardent, the skin hotter, and the phenomena from nervous derangement more severe, produce a diminution of these symptoms, softness of the pulse, and dispose to a beneficial diaphoresis; but it will be necessary to stop at the moment when the tongue, the skin, the pulse, and feelings of anxiety, give the signal of super-excitation; 3°, when the febrile period has terminated, and the patient has fallen into a state of extreme weakness, which cannot be attributed to sufferance from an inflamed viscus; that is, properly speaking, the commencement of convalescence; 4°, lastly, when no hope remains, and the congestions increase with astonishing rapidity, in spite of the employment of the most powerful revulsives. But this last case is an extremely delicate one; and I am convinced that this desperate method, to which practitioners frequently deliver themselves up too soon, has caused more victims than it has rescued patients from death. But all this must be subordinate to the sagacity of physicians; and it is always in applying physiology to the patient of disease,—that is to say, in devoting their attention, with the neglect of any exclusive system, to an analysis of the functions, to the study of sympathies, to an evaluation of the vital powers, and to an appreciation of the effects of modifying measures,—that they can acquire this necessary sagacity."

It would be easy for us to multiply quotations, and to develop still further these important considerations respecting the treatment of those severe and rapidly destructive maladies; but we should confine ourselves to an exposition of the general ideas of M. Broussais, and we have, perhaps, already somewhat diverged from our proper course.

It is a fundamental law of pathology, that all the species of irritation of which our organs are susceptible may be continued or intermittent. Periodical phlegmasiæ and neuroses have been frequently observed, and in every part of the body. These periodical irritations of the sanguineous system, when they affect other organs than the stomach and small intestines, assume characters that have obtained the name of intermittent fevers.

If the febrile diseases of a continued type, of which we have treated, are nothing else than the results of irritation of the stomach and small intestines, can these affections, when they are intermittent, be considered as idiopathic? After all that we have said respecting the causes and the symptoms of the first, we think it must be unnecessary to produce arguments in this place to prove that the second are owing to the same organic lesions. For a long time, the approximation of the symptoms of these two orders of diseases, demonstrated by M. Pinel, had led us to consider them as having the same local origin: the changes, therefore, made in the doctrine of one series must necessarily be applied also to the other. We shall confine ourselves, on the present occasion, to some reflections on the treatment of intermittent gastro-enteritis.

"When the irritation," says M. Broussais, "is so intense as to maintain the fever in a high degree, but not sufficiently so to prevent the return of fits of shivering,—that is to say, when the fever is *remittent*,—the first object should be to calm the irritation of the gastric system by antiphlogistic measures. If the periodical febrile paroxysms continue with intervals of apyrexia, the case becomes of the following species. In this the complete intermission of general irritation indicates that that of the viscera suffers a similar interruption. I say more; the paleness of the tongue, which represents the state of the mucous membrane of the stomach, the discoloration of the skin, which corresponds equally with that of the former organ, inform us that the irritation which it had suffered in the access is changed into a state of languor and asthenia. It is then that stimulants, by provoking an artificial excitement, which is transferred to the surface, prevent the return of the centripetal motion of the vital powers, which would have produced a new access. These, then, are cases for the exhibition of stimulants, which become real *tonics:* they should be administered from the complete cessation of the access to the probable epoch of its return. Three or four intervals will suffice for the termination of the fever, provided that the artificial irritation provoked be unceasingly maintained. The return of the colour of the tongue and of the skin, and a certain elevation of the pulse without uneasy sensations, are the assured presages of a degree of re-establishment of healthy action, that places the patient out of danger of a renewal of the paroxysm."

Such are the bases of the treatment which, according to M. Broussais, should be employed to combat intermittent fevers. In order that it should be followed by the expected success, the state of apyrexia should be complete; without which, the stimulants termed *febrifuges*, acting on organs in a state of irritation, would have no other effect than that of exasperating the evil, and rendering the irritation continued. It is necessary, also, that the viscera be free from chronic inflammation: this lesion, indeed, is a cause which solicits incessantly the vital powers, and constantly provokes febrile concentration. What is then the effect of excitants? They give increased activity to the irritation which produces the fever, and the vital powers continue to be exhausted. Stimulants are then neither *febrifuges* nor *tonics*; for these qualities, properly appertaining to medicines which destroy fever and raise the vital energy, cannot agree with substances which support the one and accelerate the fall of the other.

When the fever is prolonged, either because it has not been opposed, or because the excitants, applied to the organs actually the seat of irritation, have supported the morbid state, the continued irritation of the digestive viscera terminates by producing in the annexed organs, or in the lymphatic ganglions situated behind the mucous membrane of the intestines, congestions more or less considerable in degree, that have been termed *obstructions.* It has been a question long in dispute, whether the cinchona or the fever produced the tumors which are also observed after chronic and long-continued inflammation of the digestive canal, which we shall hereafter recur to. The question has appeared difficult to resolve, by the greater number of practitioners. From the observations we have made, it is easy to perceive that sometimes the disease, and sometimes the remedy, are the productive causes, and that in all cases they depend on chronic irritation of the viscera.

When we are consulted by a patient actually suffering an access of intermittent fever, and considerable congestion in one of the important organs of the animal economy has taken place, we should combat these accidents just as though the malady was continued: leeches, sedatives, and revulsives, should be employed, according to the nature of the symptoms. But, as the prevention of future attacks is of especial importance, we should administer cinchona immediately after the cessation of that, the alarming phenomena of which we had opposed. But this is a part of the subject which it is unnecessary for us to pursue further.

We have hitherto only considered primitive irritations of the digestive canal, and those, too, which immediately produce febrile re-actions: it remains for us to examine in what manner the gastric system is sympathetically influenced by irritation of other organs, and by what means fevers are then produced.

"When the influence of cold," says M. Broussais, "determines a reflux to the inward parts of the body of the blood, and the vital powers, which exalt organic action in all the viscera, and especially throughout the great extent of the mucous membranes of the gastric cavities and the respiratory organs, this reflux is followed by fever, the duration of which is for twelve hours, twenty-four hours, or from seven to nine days, according to the degree of irritation that a more or less rapid concentration has left in the three principal viscera." Such is the idea of M. Broussais respecting the inflammatory fever called idiopathic, and which he terms (we do not well perceive wherefore) *simple fever;* for the excitation of the sanguineous system which characterizes it, is then, as in all other fevers, the result of sympathy from local irritation.

Inflammatory fever may, according to M. Broussais, be produced—1°, by slight excitation of the whole extent of the mucous membranes; 2°, by the presence of too stimulant blood, which irritates their internal surface, or even by the too great abundance of this fluid; 3°, by irritation, more or less violent, of other organs than those of the gastric system. What we had previously stated renders useless any further observations respecting the two former cases; the third alone has not been elucidated, and that will now occupy our attention.

The term *inflammatory*, or *angéioténique*, *fever*, says M. Broussais, designates an excitation of the sanguineous system, which may correspond with all local irritations; and if, in all the examples we possess of this affection, the irritated organ has not been discovered, it depends on the fault of observers, who, only attentive to cerebral pain, mistake the other cries of suffering of other parts. "*Whenever an organ is sufficiently irritated to elicit fever*," he continues, "*it never produces this, but by such irritation being conjoined with that of the heart and the mucous membranes, especially those of the gastric viscera.* To which proposition the following has been opposed:—*All the tissues formed of capillary blood-vessels are capable of producing fever, and they incite it by acting on the heart, but without necessarily affecting the mucous membranes.* This diversity of opinions appears to depend on its being assumed by M. Broussais that, in all cases, irritation, not having its seat in the mucous membranes, necessarily acts on these membranes, which in turn excite the action of the heart, and fever is then produced. The constant reality of this succession of action is by no means proved. There are cases, on the contrary, where the signs of excitation of the sanguineous system precede those of the irritation of the mucous membranes.

But the most important point of the question is to ascertain, if possible, whether it is necessary that there be at the same time irritation of the heart and the mucous membranes, in order that the sympathetic irritation of the animal economy may assume that character which is termed *fever*. M. Broussais would reply in the affirmative; and that the phenomena which take place from local irritation, when the mucous membranes are not also affected, are precisely different from those constituting fever.

The succession of action, however, assigned to these cases by M. Broussais, does not appear to be universally correct. Facts demonstrate that the excitation which constitutes fever commences sometimes in the brain, sometimes in the heart, and sometimes in the mucous membrane of the stomach. If it be slight, and confined to one or other of these three viscera, we observe sympathetic delirium, accelerated action of the heart, and derangement in the digestive function, isolated from other accidents, and, according to M. Broussais, distinct from the state termed fever. But most frequently these three organs seem to be simultaneously affected; and, in other cases, the numerous connexions uniting them soon communicate the increased action from one to the other.

This is a point of pathological physiology of great importance, and would serve to explain, according to the opinions of M. Broussais, by what mechanism the more or less severe degrees of gastro-enteritis are so frequently complicated with inflammations exterior to the digestive canal. Indeed, it is easy to conceive how the habits of body of patients, the seat, the extent, the violence of the inflammation, the causes which have produced it, &c. may produce a greater or less degree of intensity in the consequent affection of the gastric system.

All the organs being united to the nervous system and to the loco-motive agents by sympathies more or less direct, it is easy to conceive how inflammation of them may suffice to produce the symptoms of *adynamia* and *ataxy*. M. Broussais readily admits that all local inflammations may *debilitate* the subjects of them, or produce the phenomena termed *nervous;* but he considers that whenever that debility or these phenomena are accompanied with burning heat of the skin, pain in the limbs, ardent thirst, and redness with dark foulness of the tongue, they are the results of violent inflammation of the stomach and small intestines.

It is, on these data, easy to perceive what course should be taken in all external affections which are productive of the febrile state. We should always avoid to apply excitants to the alimentary canal, whether they be tonics, emetics, or purgatives; and, when the sympathetic irritation shall have become so violent as to attract the attention of the medical practitioner, the same remedies should be employed for it as if it were primitive.

We have now terminated a general view of the pathological doctrine of M. Broussais, especially that part of it which relates to the most important febrile disorders; and have, we trust, clearly developed his arguments to show that there does not exist such a state of disease as *idiopathic* fever; that all febrile phenomena are sympathetic effects of local lesions; and that those fevers termed *primitive* or *idiopathic* are owing to different degrees of irritation of the mucous membrane of the stomach and intestines. The exposition we have given will, we trust, be sufficient to excite the reflection of our readers, and to furnish them with what will enable them to discover and pursue the ideas that arise from them respecting the points of lesser importance in this system of pathology. We shall, therefore, relinquish this subject for the present, since there are so many others urgently claiming our attention; but we shall, nevertheless, resume it at a future period, with a view to the further consideration of the objections of its adversaries, and to develop some other important parts of it, especially that relative to pulmonary consumption.

We cannot, however, conclude without again observing, that, although there are few points in this doctrine which, distinctly considered, evince decisive novelty of ideas, and that much, and indeed the greatest part of it, naturally flows from what we consider to be the best of those of Stahl, Bordeu, Bichat, Brown, Darwin, Beddoes, and Jackson,—yet, there are many important original propositions in it, and the arrangement and organization of the whole evinces deeply-penetrative views, great precision of ideas, and much and judicious reflective consideration. We do not adduce the names of several later and existing authors, whose doctrines have some resemblance with those of M. Broussais, because the date of the original publication of his views will show that he cannot be indebted to them; and we must also remark, that the greater number of those who have attentively studied the *Histoire des Phlegmasies Chroniques* are inclined to believe that, notwithstanding the coincidence of opinions above alluded to, the pathological doctrine of M. Broussais is chiefly deduced from original observation and reflection.

### *An Essay on the Consequences of the more severe States of Puerperality*; by J. E. Fodéré, M.D. Professor to the Faculty of Medicine at Strasbourg.

*(Continued from page 357.)*

"The *chronic delirium*, or *mental alienation*, to which women are so subject after parturition, has long engaged my attention; and I have been more readily disposed to dwell on this phenomenon in a particular manner, because I conceive it is dependant on that state of the system which I have also indicated as disposing them to be affected with several other serious maladies. I have spoken, in my *Treatise on Delirium* and elsewhere, of the accidents of this kind which I have met with in practice, and there is no physician who has not witnessed them.*

* M. Fodéré here remarks, that it is in hospitals especially devoted to the insane that facts of this kind, being assembled, form

The medical treatment of this affection has consisted in the use of mild purgatives continued for a long period of time, issues in the neck, purgative glysters repeated several times a-day, and tepid baths; blood-letting has been but rarely resorted to. Such, indeed, is the most rational mode of treatment in the opinion of the best practitioners: but did these measures cure the disease in those cases that have terminated favourably; or was it not rather nature, aided by time, that established the equilibrium of the vital actions, and prepared the salutary terminations? I have been induced to infer the latter, by the cases that have come under my own observation. M. Esquirol continues to remark, that the subsidence of this mental alienation is indicated by the re-establishment of the lochiæ, the appearance of milk in the breasts, copious and mucous alvine dejections, the return of menstruation, sometimes by leucorrhœa, and rarely by a recurrence of pregnancy. If the existing ideas, which I believe to be correct, respecting the nature of the lochiæ and the cause of the appearance of milk in the breasts, are true, it is with difficulty that it can be admitted that these two effects can take place long after the cessation of the cause of their existence: because, of 55 cases that terminated favourably, 51 did not occur until after the lapse of four months from delivery. Now, we know that at such a period there cannot be a doubt entertained respecting the possibility of a new appearance of lochiæ and of milk in the breasts. Besides, in the patients I have seen, the lochiæ and secretion of milk took place in the ordinary manner, and one of them even suckled her infant. It may also be observed, that we every day witness some women experience the want of lochiæ, or at least only have a trifling discharge and of short duration, and others have no milk whatever, without falling into this state of mental alienation.

I conclude, from the nature of the chronic delirium of puerperal women, from the epoch at which it manifests itself, from the absence of a primitive material cause, from the class of persons most frequently the subject of it, and from the absence of any apparent organic lesion on examination after death, that this affection should be placed amongst the neuroses.

I have now arrived at the most important part of my labour,—that, indeed, for which it was almost exclusively undertaken,—I mean *puerperal fever*. Hippocrates recognized the influence of the state of puerperality on reigning diseases: he gives, in the first and third books of his *Epidemics*, the history of several parturient women attacked with fever, the course of which was evidently

---

the most striking pictures: he would, therefore, point out the observations made at the Salpetrière, by M. Esquirol, as illustrative of the most common causes and consequences of this affection; but, as those observations have been already detailed in the 238th Number of this Journal, I shall pass on to the author's reflections on the remedial measures usually adopted in those affections.—Translator.

different from the reigning malady, and of which the favourable or fatal termination could usually be judged of on the fourteenth day.* The second, fourth, fifth, sixth, and seventh books, although written by different authors, furnish also examples of it, but their history is less complete.

Notwithstanding the descriptions of the Father of Medicine, which are extremely faithful, and of which I have a striking example under my observation at the present time, too little attention has been paid to this state of puerperal women, in whom little more has usually been attended to than the retention, or too copious flow, of the lochiæ, and the different accidents dependant on the secretion of milk. Sydenham himself did not extend his views much further: he merely remarked, that the fever which sometimes follows the suppression of the lochiæ, might pass into the class of the reigning maladies; and that it should then be treated like the epidemic, considering, however, the particular state of the subjects of the disease. This opinion has been generally followed even by Boerhaave, who added nothing to it in that part of his *Aphorisms* relative to puerperal women. It could not, however, have escaped the observation of these great physicians that women in this state are sometimes attacked with fever, independant of retention of the lochiæ and of any reigning epidemic. The mortality that took place among them, in 1746, at Paris, described in the *Memoires de l'Academie des Sciences*; and the malady that made such ravages in London and several other parts of England in 1768 and several ensuing years, which thence received the name of *puerperal fever*; finally, that which we now daily observe,—proves too effectively that there then exists a peculiar pathological state, the treatment of which is difficult, the prognostic obscure, and the issue doubtful, which has not been classed in our modern nomenclatures; but of which it is important that young physicians should not be ignorant, or disdain to admit, on commencing their professional career.

This fever, when it occurs, commonly manifests itself, as Hippocrates observed, on the second or third day after delivery, which is also the period at which *milk fever* usually appears. Sometimes, however, but more rarely, it shows itself considerably later, even as distant as the forty-fifth day; and sometimes also shortly before delivery. The patient is suddenly seized with shivering and horripilation; to which succeed heat and dryness of the skin, sometimes moderate, sometimes very intense; cephalalgia; inquietude; contracted pulse, at first not much increased in frequency, sometimes very full, at others presenting a sort of vacuity, and extremely variable on the slightest adventitious causes of disturbance, in consequence of the extreme sensibility of body and mind.

---

* The author here adduces several of the cases to which he alludes; but I pass them over, considering that the works of Hippocrates are, or ought to be, familiar to every student of medicine.—Translator.

This coincidence with milk fever sometimes prevents sufficient attention being paid to puerperal fever on its origin, which does not differ from it, in the greater number of cases, in the first instance, but in the sensation of weakness and vague pains in the abdominal region of which the patients complain; symptoms which are, however, not yet strictly characteristical. These pains soon become fixed, and are, for the most part, attributed to the hypogastric region, which becomes swelled, tense, and very tender to the touch. There is a great degree of lassitude, and heat and dryness of the skin, which are not followed by the vapour from the surface that is witnessed in milk fever. The essential symptoms are—weakness, inquietude, lassitude, pain within the abdomen, and a temperature of the skin constantly above the natural standard. The tumefaction and tension of the breasts, and the painful sensations, extending to the axillæ, which thence result, are not always sufficiently remarkable to admit of the inference that, from their absence, we may consider the existing fever as that properly termed puerperal; or, from their presence, that the existence of this should be denied: and, when the phenomena of milk fever, of peritonitis, and of metritis, are combined, as sometimes occurs, it is only by the greater degree of violence, the extension, and the continuity, of the symptoms, that we can really perceive, in its early stage, that there exists a malady more severe than the milk fever.

There is sometimes constipation of the bowels, in the first instance; but this is soon followed by an abundant flux of bilious, brown, very fetid, matter, with tension of the abdomen, and distressing flatulence. Nausea and vomiting are frequent symptoms; the urine, when it is not coloured by the lochiæ, will be found to be limpid and crude; and there is sometimes dysuria. It should be here remarked, that the vital powers fall so much within the first few days of the disease, that the physician experiences some consternation on witnessing the rapid change that has taken place in the physiognomy of the patient: he sees anxiety and sadness forcibly pourtrayed; the eyes are sunken and dull, or else they have a sort of brilliancy that leads to fearful presages. The patient utters deep and prolonged sighs; she is discouraged, and disposed to predict the termination of her existence; there is constant thirst; short, difficult, and oppressed, respiration; a dry cough; a globular or spherical prominence of the abdomen; the forehead (particularly about the eye-brows), the shoulders, the hips, the thighs, and the loins, are painful; there is also pain experienced, especially about the navel or epigastrium, under the false ribs, between these and the groins, and in the region of the bladder: this pain, sometimes after a remission, soon becomes more acute, and is increased on the slightest pressure. Some women, however, only experience pain, tumefaction and tension of the abdomen, and difficulty in respiration, towards the termination of the disease; others are seized with pain, swelling, and tension of one or other of the thighs, which deprive them of the power of moving it, and a critical abscess is formed in this part, which sometimes appears to be connected with the alleviation of the malady. Some women, who have had a considerable quantity of milk, have the breasts suddenly become lax and shrunken; in others, these organs remain firm and prominent until the disease is about to terminate fatally, when they sink from the effects of the fever and diarrhœa. It is the same with respect to the lochiæ, which certainly are not suppressed in all cases of what is called puerperal fever. When the disease is less severe, and is disposed to terminate favourably, the patients experience relief from diarrhœa, increased flow of urine, and cutaneous perspiration; which are accompanied with a favourable change in the disorder at some period between the seventh and fourteenth days. When it terminates unfavourably, continual agitation and inquietude; frequent fits of fainting; delirium, without which I have, however, seen several women die; drowsiness; hiccough; spasms; convulsions; sometimes loss of voice; copious sweats; pains like that from pleurisy; great anxiety; and, if the period is continued for a long period, there are sometimes remissions which excite hope of its favourable termination, the patient being one day better than on another, but a recurrence of its former severity again places the patient in the same alarming state. Sometimes a flow of acrid and eroding serum takes place from the vagina, without being followed by any amendment; the joints of the wrists, elbows, knees, and ankles, became covered with erysipelatous inflammation, of a reddish-brown colour, and in patches of the size of a half-crown piece, according to the observations of Home and Denman, but which I have not myself ever witnessed; the pain within the abdomen continues to increase, and disappears only a little before death, and the patient voids unconsciously urine and fecal matters of an extremely offensive odour; the pulse continues to sink; the tongue and the extremities become cold; and, lastly, convulsions, or a comatose or apoplectic state, terminate life during a febrile exacerbation.

The course of this malady is frequently very rapid, terminating in death within twenty-four hours. Denman mentions having seen some cases in which the state of coldness was not followed by heat; and others in which death happened unexpectedly after an almost insensible progress of the disease, and without apparent indications of danger: a lady of Strasbourg has recently died under similar circumstances. When the symptoms proceed with less rapidity, death commonly takes place about the eleventh day; but in some instances life is prolonged till the third week, a month, forty days, or longer. We should especially fear it when the disease has manifested itself either before or shortly after delivery, and when the progress of severe symptoms is very rapid. Diarrhœa occurring immediately after the attack of the disease, and spontaneous vomiting, are sometimes critical, particularly when copious stools are followed by a subsidence of the abdomen and moisture of the skin. Abundant and universal sweats succeeding to paroxysms of cold are a good sign; a free evacuation of the lochiæ is also a favourable indication; and we may also cherish hope when the pulse, after having been quick and small, becomes more slow, soft, and full; when the respiration remains free; when the lochiæ reappear after having been suppressed; when the urine deposits a thick, grey, white, or even brick-coloured, sediment; and when the patient, from time to time, enjoys tranquil sleep. In some desperate cases, from which the patients have nevertheless recovered, it appears that they have owed their escape to a constitution sufficiently strong to support the long duration of the flux from the bowels, and also from their having previously somewhat recovered from the effects of labour.

I shall now examine distinctly some symptoms and signs which are not precisely similar in all cases, because of the particular constitution of the patient, the part affected, the degree of intensity of the disease, and the epoch of its invasion after delivery, give rise to several varieties. I shall afterwards point out to the young physician the degree of importance that should be attached to certain symptoms and changes, in order that he may neither experience alarm himself, nor give rise to dread in others, without appropriate reason: I shall consider the pulse, the temperature of the body, the tongue, the indications of the state of the stomach, certain cutaneous eruptions, griping of the bowels, and the effects of spasm.

The pulse, in certain cases, is merely frequent and contracted, sometimes full and almost natural, or hard and strong, like what is observed in inflammatory affections in persons of a robust habit; but, in other cases, and these are the most common, there is almost always, from the beginning, an extraordinary quickness of the pulse; sometimes, too, it is quick and much more feeble than might be expected from the concomitant circumstances. But, whatever it may be at the commencement, it always becomes very frequent on the approach of a fatal termination of the disease; and Denman correctly observed, that we rarely witness a more unfavourable sign in this fever than that furnished by great acceleration and weakness of the pulse, although the other symptoms may appear less prominent.

The heat of the skin is ordinarily attended with an arid state of it; sometimes there are copious, but partial, sweats; in some cases, the heat always remains the same in degree; in others, there is one or more attacks of shivering during the day, or these occur every other day. An icy coldness sometimes precedes death for about twenty-four hours: it is a sign of the existence of gangrene.

The tongue is ordinarily dry; sometimes it is of a greenish-yellow colour about its base; it differs, however, in appearance according to the progress of the disease, and even sometimes, although the case be severe, it is but little altered; it occasionally continues moist, and becomes covered with a thick white coating.

The taste is also generally altered, and many patients present signs of an inflammatory state of the stomach. In all cases, unless these signs have preceded delivery, we should not be easily led to consider the disease merely as bilious or mucous gastric fever. Indeed, we see women after parturition, in other respects very healthy, suddenly display the appearances of this fever, without having previously shown any sign of derangement of the alimentary canal; and we know that nausea and bilious vomiting are often merely effects of inflammation of some one of the organs seated in the lower part of the abdominal cavity. But, according to the reflections I have already adduced respecting the derangements of the digestive system induced by pregnancy, it will be evident that these signs will sometimes indicate an original, and not a merely symptomatic, disease; and they will also show that they should always engage our assiduous attention.

We sometimes observe petechial eruptions in the first stage of this disease; but this is not an essential occurrence, and it depends either on its complication with some other disease, or on certain states of the air or unhealthy situations. The most frequent species of eruption is the miliary, which is also commonly observed in mucous fevers, and consequently does not particularly relate to puerperal women. This symptom, which is rarely met with in the south of France, very frequently shows itself in the moist valleys of Piedmont, of Alsace, of Franche-Comté, and along the lower Rhine; as well as in a long-continued, either hot or cold, moist constitution of the atmosphere: it has very improperly been attributed to the method of treatment. I have met with it in ordinary mucous fevers, without finding any particular consequences ensue from it; but an ancient prejudice causes the disappearance of it to be regarded as fraught with danger to puerperal women, and I believe that this opinion ought to be respected. In the year 1790, when I practised in the valley of Aoste, I was called to visit, in the town of Verres, a young woman in childbed, residing on the side of a causeway, who was covered with a miliary eruption. All her attendants were in a state of great alarm: I, on the contrary, a partizan of the adversaries of Professor Allioni, who had published a book on this subject, to say the truth, a little biassed,—I endeavoured to assure them of her safety, since her other symptoms were not alarming. Two days afterwards, passing that way, on making enquiries respecting my patient, I found she was buried.

The griping pains which women recently delivered suffer, nearly resemble those which accompany puerperal fever: they are sometimes even more insupportable than those of labour; but they may be distinguished by the regular course of the other attendant circumstances, and by the intervals of a total absence of pain in the first case; whilst this exists constantly in the latter, notwithstanding the remissions of the fever. We should be also attentive to the returns of shivering, of pain and uneasiness; and it is on the recurrence of these only that we should begin to experience alarm.

The absence of the lochiæ, and the signs of abdominal inflammation, often depend, in weak and delicate women, merely on a state of spasm and erethism of the vessels of the uterus, and only require the exhibition of opiates and antispasmodics. They may also be produced by the presence of irritating matters, by clots of blood, or by portions of the placenta remaining in the uterus. Moderate evacuants and mucilaginous injections soon dissipate these symptoms, whilst they would certainly be exasperated by the treatment appropriate for puerperal fever, should they be mistaken for it. We should always pay due regard, when the labour has been of rather long continuance, or the woman is of a particularly nervous habit, to the violent agitation of the whole vascular and nervous systems, which produces such a degree of celerity of the pulse, a general excitation, constant thirst, and great inquietude, that a person not familiar with this state would be inclined to believe in the existence of an ardent fever: but calmness soon ensues after a little repose in bed, the flow of the lochiæ, fomentations, oily and antispasmodic potions, and emollient glysters. It is only when this state obstinately continues after the first or second day that the experienced practitioner presages something out of the ordinary way.

Dissections of the bodies of the victims of puerperal fever, which have of late years been made to a great extent, have most frequently shown the traces of inflammation of the peritoneum and its diverse productions: sometimes inflammation of the uterus, the fallopian tubes, the ovaries, and the intestines. Collections of thick, yellowish-coloured, serum and purulent matter have also been most frequently observed, with coagulated lymph, resembling curds, floating in the abdominal cavity, and in part agglutinated to the intestines, to the peritoneum, the epiploon, or the uterus; frequently also there have been found in the same subjects similar effusions of serum into the cavity of the pericardium and thorax, and collections of lymph on the pleura, the lungs, and the pericardium.

The above appearances, as I have stated, have been most frequently witnessed; but these signs of inflammation of the membranes about the uterus have not always occurred. When death has arrived with great rapidity, nothing has been observed, except a considerable quantity of serum within the pericardium, or in the cavity of the thorax.

Admitting, however, since such is frequently the case, that inflammation of the peritoneum accompanies puerperal fever, it will still be necessary to ascertain if this inflammation be primitive or secondary. There can be no doubt but that the former species of inflammation may take place, as a consequence of the causes that produce peritonitis in persons in general; and Dr. J. P. Franck even asserts that this is sometimes epidemic: it is then especially so amongst puerperal women, from their particular disposition to it; but there is this difference when these are the subjects of it, that although it is neither of a very serious character, nor difficult of treatment, in persons in general, in lying-in women, it sometimes displays the most prompt and alarming symptoms, and carries them off during the first or second day, and before any decisive mode of treatment can be put in practice: whence it is evident that other circumstances unite in puerperal women to give to this inflammation a character and a degree of distinctiveness different from the ordinary forms of that affection. Does not the most simple exercise of reason inform us, that the inflammation accompanying the malady which is the express subject of my Memoir has nothing in common either with the causes of this state of disease, or the ideas to which it gives origin?

To be convinced of the difference that exists between primitive peritoneal inflammation and the true puerperal fever, we have only to pay due attention,—1°, to what happens when that inflammation exists independantly of puerperal fever; 2°, to the phenomena of metritis, certainly an acute form of inflammation, but which I have seen go through its course without the symptoms of puerperal fever, and which is also a much less severe disease, and more easily alleviated, than the latter.

The true peritonitis is similar in its terminations to other inflammations under ordinary circumstances, and may, like those, be either acute or chronic. I have seen many women in whom this inflammation, accompanied with acute or dull pains and dysuria, has been very manifest at the termination of labour, in whom suppuration has ensued from the inflammation, and who have nevertheless recovered; a success which I certainly, however, would not venture to boast of.

It has been long known, and been said to relate to real puerperal fever, that the matter from suppuration of the peritoneum sometimes collects about the navel, and is evacuated by an opening in that part; a circumstance that should be favoured by all appropriate means, and particularly by a proper situation of the body. The *Mémoires de l'Academie des Sciences*, for the year 1728, furnish examples of it related by Chomel, and similar occurrences are also described by Levret. It will, nevertheless, be more prudent not to wait for these natural efforts under those circumstances, but to procure an artificial evacuation by means of the cautery, in the situation where the greatest tumefaction and fluctuation are observed, taking care at the same time to sustain the strength of the patient by cinchona and analeptics; a method which I cannot but approve of, when employed after the violence of the inflammation has subsided. Now, who can isolate these cases from the idea of peritonitis, and say that this inflammation and puerperal fever are one and the same disease?

Metritis, as we have already stated, although it may apparently evince some analogy with puerperal peritonitis, in reality differs from it in many important circumstances. The inflammation of the uterus more decidedly evinces itself; it is induced by more manifest causes,—as violence of some kind, a wound, contusions, compression, extraneous substances, too great distention, forced contractions, violent and long-continued efforts, &c. The fever which accompanies it is more characteristic of inflammation, and analogous with that attendant on inflammation of other viscera: this fever is, indeed, announced by more intense cold, with considerable shivering and inquietude, to which succeed burning heat; full, hard, and frequent pulse, which becomes in the end small and weak; fixed pains, like common after-pains, appearing to descend from the loins, subsiding after a little time, but shortly returning with more intensity. The vomiting, and many other general symptoms, are common to metritis and peritonitis; but the tumefaction of the abdomen is very different, and, notwithstanding the consent between the peritoneal envelopments of the uterus with the rest of that membrane, it is rare that the swelling becomes extended, except in fatal cases. The tumefaction does not manifest itself so soon as in puerperal peritonitis: it is more circumscribed; it answers to the figure of the uterus; it may be felt above the pubis, about the middle of the abdomen, where the patient experiences a sensation of great heat; the pain often extends to the neck of the uterus, to the orifice of this organ, and to the vagina, which never happens in puerperal peritonitis. The milk and lochiæ disappear also more constantly in metritis. Now, since so severe an inflammation of the uterus differs so much from puerperal fever, it is evident that it is not its inflammatory character that forms the essential circumstance of the latter affection."

*(To be continued.)*

---

*Case of Rupture of the Heart, without having been preceded by Lesion of its Structure;* by Dr. Fischer, of Hildburghausen. [From the *Journal der Praktischen Heilkunde.*]

Although there are several cases on record of rupture of the heart, in which it has been supposed that previous alteration of its structure had not existed, yet the case here related by Dr. Fischer must be contemplated with considerable interest, as there are some remarkable circumstances attendant on it, and such as appear to point out the mode in which that pathological phenomenon took place in this, and, perhaps, the generality of analogous cases. We shall give an abstract of the history of the case given by Dr. Fischer, which is very long, and is, indeed, extended by many minute observations which we consider may be omitted without detracting from the utility of the narration.

The patient was a man 68 years of age, who, after having long resided at court, was obliged to quit it and live in the country. He soon after this was affected with indigestion, and various nervous disorders, and he became a prey to the most sad moral affections; but he concealed with so much art what passed in his own mind, that, with the exception of his family and the physician he consulted, every one supposed him to be resigned to his fortune, or to be insensible to his disgrace. The slight paroxysms of gout, to which he had previously been subject, now occurred less frequently, and appeared at most only on remarkable changes of season. His mode of life did not tend in other respects to injure his health.

On the 16th of October he was seized, whilst walking, with a violent pain, which he considered to be spasm of the stomach, and he with extreme difficulty reached the house of a friend, at the distance of half a league. He there threw himself into an arm-chair, and could hardly get breath. Some of Hoffmann's anodyne was given to him; soon after which he recovered, and remained in the society of his friends, without making any complaint, until his daughter arrived with his carriage to take him home. He passed a good night after this, and hardly felt himself indisposed. The following day he went to the same place which he had visited on the preceding evening; and, on returning thence on foot, accompanied by a servant, he, after having walked about one-third of the distance, was attacked with the same paroxysm of pain: he was above an hour in walking the remainder of the way; but, on getting home, he soon recovered, supped, went to bed, and slept very well. The following day (October 18) he wrote to Dr. Fischer, to inform him of the above circumstances, and to request him to prescribe somewhat that would relieve him from the wind in his stomach, which he considered to be the cause of his disorder. Dr. Fischer, however, thought differently of the nature of his complaint, and advised him to take a mixture composed of Hoffman's anodyne, volatile liquor of hartshorn, extract of carduus benedictus, and infusion of valerian; which he took. The patient felt quite well during the ensuing evening and following morning; but on that day (October 19), on sitting down to write after coming from church, he was seized with a general shivering and spasmodic contractions of the extremities, accompanied with dreadful and almost intolerable pain in the stomach. Dr. Fischer saw him an hour and a half after the commencement of this attack: a little pain about the pit of the stomach only remained at that time; the region of the abdomen between the pit of the stomach and the navel was still very sensible to the slightest touch, but it was neither tense, hard, nor tumefied; the temperature of this part, as well as that of the face, was natural,—the countenance was, indeed, a little redder than ordinary; but the extremities were cold, and the skin of them dry and somewhat rough. The pulse was nearly natural, only now and then there were two or three irregular intermissions. The patient had taken some soup since the attack, and felt disposed to take more food. Dr. Fischer considered that it was a gouty crisis, and ordered the patient to keep in bed. He prescribed some diaphoretic medicines and anti-spasmodic glysters. In the afternoon he was apparently better; only suffering inconvenience from eructation of wind from the stomach, with which he had from the commencement of the disease been troubled. Nothing particular occurred after this until the 20th of June, when he experienced another paroxysm early in the morning. Dr. Fischer arrived at ten o'clock, and found him on the decline. A new symptom appeared in this attack,—a sensation of distension of the whole left arm, extending to the extremities of the fingers. At half-past eleven he was tolerably calm: when Dr. Fischer left him. At two o'clock Dr. F. received a message, informing him that the patient had vomited much bilious and mucous matter, which was followed by considerable relief. Another messenger immediately arrived to request his attendance with all possible expedition. On this occasion Dr. Fischer witnessed the paroxysm nearly from its commencement to its termination; and he states that he had never seen any thing so horrible. Two strong men were leading, or rather dragging, the patient about his room; there was extreme agitation of his whole body; despair was depicted on his countenance; his cries were a sort of scream; and he fervently invoked the arrival of death. By urgent entreaties he was persuaded to lie down: some salt of hartshorn and opium were given to him, with some strong infusion of chamomile; and a cataplasm of henbane and belladonna was applied to the region of the stomach. The skin soon afterwards became moist, the pain diminished, and the patient experienced shooting sensations sometimes in the great toes, sometimes in the right and in the left shoulders; and the pain of the stomach now and then momentarily disappeared. Assafœtida, alternately with the salt of hartshorn and opium, were administered; and eructations of wind from the stomach took place occasionally with sensible alleviation. The patient after this no longer complained of his stomach, but said the pain, although less violent, seemed to be spread throughout the whole of the body. In proportion as the extremities regained their natural heat, the pulse, from having been small, contracted, and intermittent, became more free; and, when a sweat broke out, it was found to be nearly regular and natural. This sweat was very copious, and the patient again experienced some degree of pain in the stomach: he was, however, calm; and Dr. Fischer left him with the hope that he should witness more decided amendment; but the following day he received information of the death of the patient. The following account was given to him:— About an hour after the departure of Dr. Fischer, the pain increased, and the sweating continued to an equal degree. The patient suddenly raised himself on his seat, looked wildly round, seized his nurse violently by the neck as if he would have strangled her: "Support my head with your arm," he cried. He remained in this position for more than half an hour; after which he laid down on his left side, then on his right, and expired without a struggle.

*Examination of the dead Body.*—Externally, the body was observed to be of a bright vermilion colour over the whole of the posterior part, even down to the heels. The situation of the stomach and colon deviated from the ordinary state; the cardiac orifice was in the usual position, but the stomach thence extended upwards to the left under the arch of the diaphragm, so as to be situated very high and wholly on the left side. The ascending portion of the colon rose, from the extremity of the cœcum, forming an obtuse angle, as far as the arch formed by the stomach, the direction of which it followed in close attachment to it: it afterwards descended, forming an acute angle. The thoracic viscera were in the ordinary situation. The pericardium was covered with a layer of fat: on separating this, it was found to be much distended, and appeared to contain a substance of a deep-blue colour. On making an opening into it, three pounds of bright-red blood escaped; and, on entirely laying open the pericardium, the heart was found to be surrounded with a large quantity of clotted blood. A rupture of the left ventricle was then discovered: this extended from the point towards the auricle, was an inch and a half in length, and two-thirds longer on the external than on the internal surface of the substance of the heart; the edges of the opening were fringed. This organ, in other respects, did not show any sign of disease; there was nothing apparent that indicated any other alteration of its structure.

After referring to numerous analogous cases, related by German, French, Italian, and English, physicians, Dr. Fischer enters at some length into the consideration of the nature of the phenomena witnessed in that here related. But, as all that is adduced on this point is solely conjecture, we had, perhaps, better leave our readers to form their own opinions respecting them. We may, however, briefly state that Dr. Fischer considers that the rupture of the heart took place gradually, and that it commenced when the patient had the first paroxysm of pain. He attributes the supposed spasms of the stomach to the violent distension of the muscular fibres of the heart; and he explains the recurrence of the paroxysms to the occasional causes to which the patient was exposed on these occasions: as walking against the wind; the shaking of a rough carriage on a stony road; and, lastly, the efforts he made to sing in a loud tone of voice in church. The rupture he supposes, from its being more extensive externally than internally, to have commenced from without, and extended inwards during each paroxysm; until, on the most internal fibres giving way (probably when he started up in bed and grasped the nurse), effusion of blood into the pericardium, and death, ensued.

---

